# Artificial intelligence in retinal disease: clinical application, challenges, and future directions

**DOI:** 10.1007/s00417-023-06052-x

**Published:** 2023-05-09

**Authors:** Malena Daich Varela, Sagnik Sen, Thales Antonio Cabral De Guimaraes, Nathaniel Kabiri, Nikolas Pontikos, Konstantinos Balaskas, Michel Michaelides

**Affiliations:** 1 UCL Institute of Ophthalmology, London, UK; 2 Moorfields Eye Hospital, London, UK

**Keywords:** Retina, Artificial intelligence, Age-related macular dystrophy, Inherited retinal disease, Diabetic retinopathy

## Abstract

Retinal diseases are a leading cause of blindness in developed countries, accounting for the largest share of visually impaired children, working-age adults (inherited retinal disease), and elderly individuals (age-related macular degeneration). These conditions need specialised clinicians to interpret multimodal retinal imaging, with diagnosis and intervention potentially delayed. With an increasing and ageing population, this is becoming a global health priority. One solution is the development of artificial intelligence (AI) software to facilitate rapid data processing. Herein, we review research offering decision support for the diagnosis, classification, monitoring, and treatment of retinal disease using AI. We have prioritised diabetic retinopathy, age-related macular degeneration, inherited retinal disease, and retinopathy of prematurity. There is cautious optimism that these algorithms will be integrated into routine clinical practice to facilitate access to vision-saving treatments, improve efficiency of healthcare systems, and assist clinicians in processing the ever-increasing volume of multimodal data, thereby also liberating time for doctor-patient interaction and co-development of personalised management plans.


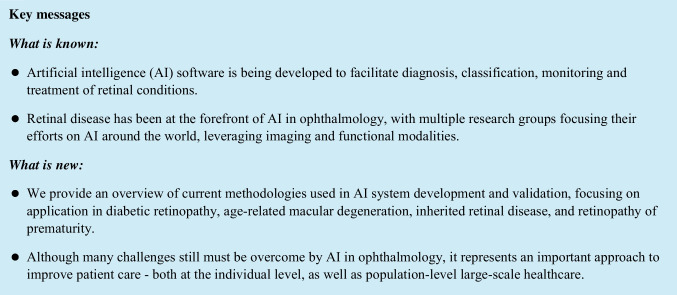


## Introduction

Retinal diseases are a significant cause of visual impairment and blindness, both in adults (secondary to age-related macular degeneration (AMD) and diabetic retinopathy (DR)) [1] and in children (due to inherited retinal disorders (IRD) and retinopathy of prematurity (ROP)) [2]. Diagnosing these conditions usually involves multimodal testing and multiple consultations with retina specialists, often not available in a timely manner, which can result in delays in sight-saving treatments. For rare diseases, it can take several years for a final diagnosis (‘diagnostic odyssey’), resulting in uncertainty about the prognosis and delay in appropriate care.

Healthcare data increases by approximately 50% every year, making it one of the fastest-growing digital areas [3]. Genomic data alone is as demanding in terms of data acquisition, storage, distribution, and analysis as astronomy or social media content [4]. Ophthalmology is one of the leading data generators, with 30 million optical coherence tomography (OCT) scans performed yearly in the USA [5]. This ever-increasing vast amount of data, alongside the development of cutting-edge digital technology, has made ophthalmology a pioneer in digital innovation and healthcare artificial intelligence (AI).

AI has been rapidly developing in multiple areas of medicine, including, dermatologist-level performance at detecting skin cancer [6], highly accurate classification of pulmonary tuberculosis [7], and genetic variant calling and classification [8]. AI-based ophthalmology telemedicine has been beneficial during the COVID-19 pandemic [9], and remote evaluation and analysis of retinal imaging may be useful in decreasing diagnostic time and facilitating triaging and classification [10, 11].

Development of highly sensitive and sensible AI-based tools requires transdisciplinary collaboration between clinicians and software engineers. Herein, we will provide an overview of current methodologies used in AI system development and validation and focus on clinical application in prioritising retinal diseases.

## AI methodology overview

**Fig. 1:**
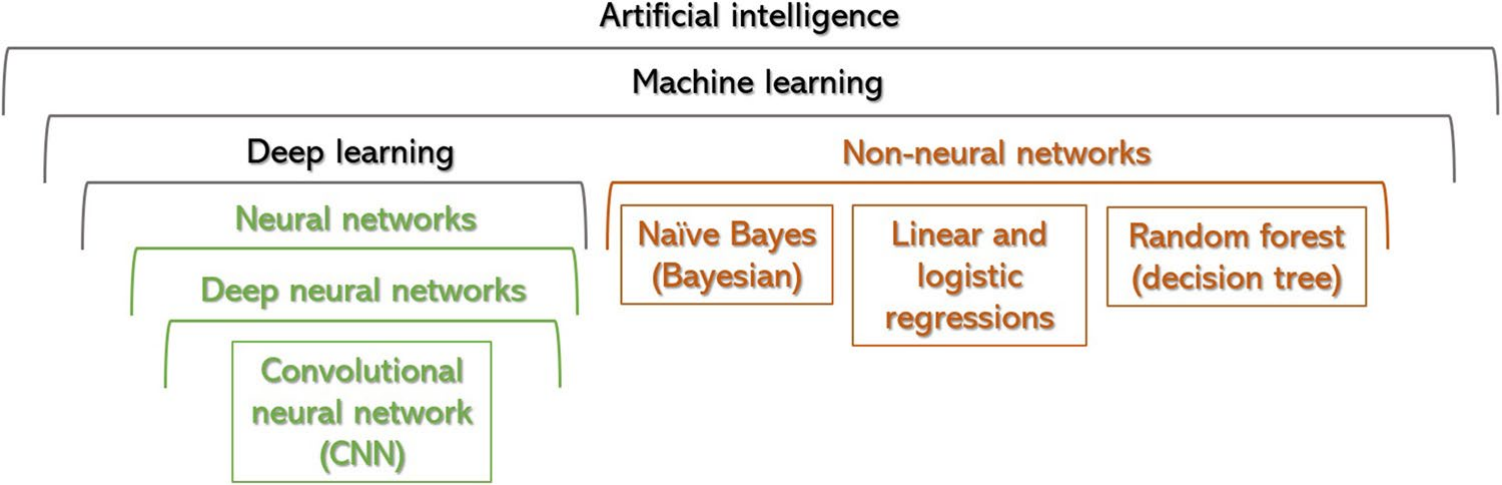
Diagram of artificial intelligence algorithms, subfields, and mechanisms

**Fig. 2:**
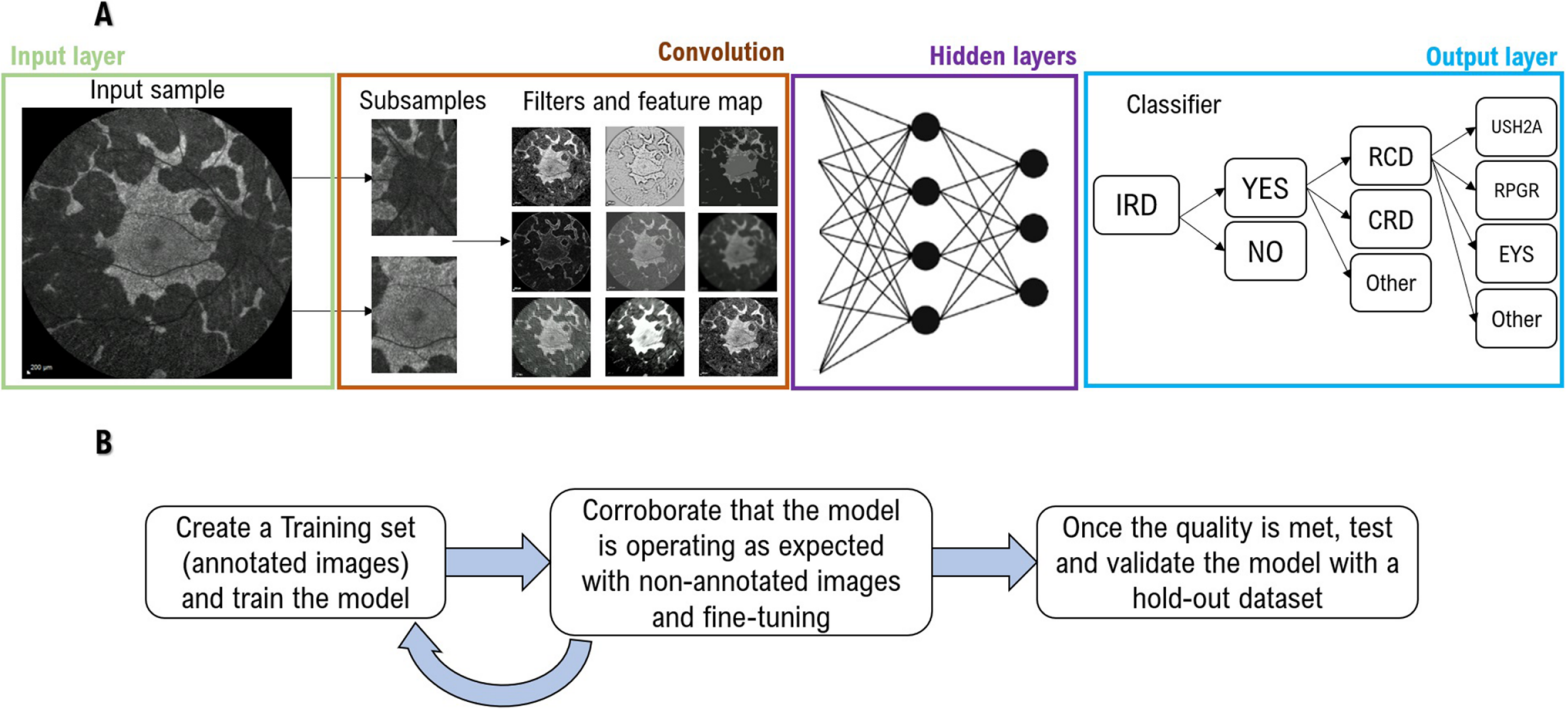
**A** Overview of a convolutional neural network (CNN). The process starts with an input layer, typically an image or video, that gets divided into subsamples and/or pixels and is analysed by multiple convolutional layers that filter, mask, or multiply features and feed the results to a dense neural network of multiple nodes ‘artificial neurons’. Each one represents a characteristic to be analysed (e.g., pixels, diagnoses, age, contrast, etc.) and is connected to hidden layers that sum and analyse all inputs, combining the received stimuli and designing a new one, leading to an improved output layer and final diagnosis. **B** The process of developing a supervised AI model. First, a training set needs to be created, and these images are used to train the model to interpret the different features; after this, a separate, non-annotated dataset (validation set) is presented to the model to try it, whilst still fine-tuning its configuration; and lastly, the algorithm is tested on new data, evaluating its overall performance

The most common techniques to develop AI-based healthcare tools will be summarised below and in Figs. 1 and 2:

- AI is a phenomenon in which non-living entities mimic human intelligence [12]. It is an umbrella term encompassing a spectrum of computing programs. ‘Rule-based’, ‘hard-coded’ or ‘symbolic AI’ has existed for many decades and is the basis of any software system, from a traffic light management system to the autopilot flying every plane. In healthcare, symbolic AI has multiple applications, e.g. calculating cardiovascular risk index or eGFR.
- Machine learning (ML) is an AI subfield in which a program achieves a task by being exposed to vast volumes of data and gradually learning to recognise patterns within the data, allocating data to distinct classes [13]. It involves ‘soft coding’, which means that the model learns from examples instead of being programmed with rules [12]. ML models can be supervised (based on data labelled by humans), unsupervised (i.e., grouping features within categories), or reinforcement learning (the system accumulates its own feedback to improve through a reward function) [14]. In medicine, supervision is the most common.
- Nonneural network-supervised ML algorithms are useful in healthcare for prediction modelling and evaluating associations and best-fitted lines between two (linear regression, parametric) or multiple variables (random forest, non-parametric). The latter combines different inputs using a network of flowcharts (known as decision trees); each tree creates an outcome, and a collective one will be made by combining all the singular outputs [15]. Non-neural networks are often combined with deep neural network (DNN) architectures and achieve improved performance (Fig. 1) [16].
- Deep learning (DL) is a subdivision of ML, defined by the presence of multiple layers of artificial neural networks (ANN) [17]. An ANN is composed of an input layer of multiple nodes—‘artificial neurons’—that represent characteristics to be analysed, e.g. pixels on an image, diagnoses (International Classification of Disease (ICD) coded), age, nucleotide changes, etc.; connected to one or more hidden layers that sum and analyse all inputs, and transmit a final value to an output layer (Fig. 2A).
- DNN corresponds to multi-layered DL algorithms (with often over 100 hidden layers), which are currently the gold standard for image classification [15]. As more layers are added, an iterative training phenomenon starts occurring, by which deep layers combine stimuli sent from other layers and design new stimuli, improving the output layer and ultimately leading to better diagnoses [8].
- Convolutional neural network (CNN) is a type of DNN particularly useful for image and video analysis [15]. These algorithms divide the files into pixels, convert them into numbers or symbols, analyse them by multiple convolutional layers that filter, merge, mask, and/or multiply features, and feed the results to a dense neural network that will create an output layer [18]. Fully convolutional networks (FCN) feed the output layers themselves, without the final step of dense layers (Fig. 2A) [17].

## Useful concepts to better understand AI literature

There are different types of models, depending on the outcome prediction. (i) Classification models apply to categorical outputs, such as classifying retinal images into with or without DR; (ii) segmentation models are specialised for image processing and analysis, detecting presence or absence of features (e.g., intraretinal fluid), segmenting images into known anatomical correlates, or classifying them into diagnostic categories; (iii) regression models for when a quantitative output is needed, such as predict central macular thickness from an OCT file [19, 20].

Different performance metrics are used to present results of each model type. Dice similarity coefficient (dice score) and intraclass correlation coefficient (ICC) are metrics of segmentation accuracy suitable for evaluating performance of image segmentation DL algorithms, ranging from 0 to 1 [21]. There are multiple performance metrics for classification and regression model algorithms, such as (i) receiver operating characteristic (ROC) curves, that plot true positives (sensitivity) against false positives (1 = specificity) [22]; (ii) the area under the curve (AUC, also known as AUROC) ranging from 0 to 1, with 1 indicating a perfect algorithm [23]; (iii) precision-recall curves (PRC), which associate positive predictive value with sensitivity (also known as recall or true positive rate) [24]; (iv) the accuracy statistical score; (v) absolute difference; (vi) Pearson’s correlation between said parameters (the latter also goes from 0 and 1) [23].

The process of developing a supervised AI model generally involves three stages: (1) training, when the network is provided labelled images; (2) fine-tuning, where the model starts aiding the manual annotation and human graders to correct it and improve it; (3) validation or testing, where the algorithm is tested on a hold-out dataset annotated by human graders and kept separate from the training dataset (internal validation). External validation on datasets of completely independent origin than the training dataset is the gold standard for validation/performance evaluation, indicating generalizability (Fig. 2B). [23]

## Selected retinal diseases for which AI-based tools have been developed

### Diabetic retinopathy (DR)

Recent studies have shown that AI-based DR screening systems can achieve adequate levels of safety [25–29]. These algorithms include classical expert-designed image analysis, mathematical morphology, and transformations [30–33]. One of the approaches tested was to classify colour fundus images from training datasets into referable DR (moderate or advanced stage) or non-referable DR (no or mild DR, Table 1). These studies either built their own CNNs or used pretrained ones like AlexNet [34], Inception V3 [35], Inception-Resnet-V2 [36], and Resnet152 [37]. Other studies tried to detect DR based on fixed features such as red lesions [38,39], microaneurysms [40], exudates, and blood-vessel segmentation [41, 42]. Lastly, other groups introduced a method to detect DR and diabetic macular oedema (DMO) using a CNN model, being able to detect the exact stage of DR; these studies are summarised in Table 1 [49–60, 95].

**Table 1:** Artificial intelligence in retinal disease—methods, cohorts, and overall results

Condition	Imaging analysed	Database (n)	AI tool	Task	Performance (metrics provided by each paper)	Publication
DR	Colour	DiaretDB0 (130), DiaretDB1 (89), and DrimDB (125)	CNN	Referable/non-referable DR	Accuracy 99.17% (DiaretDB0), 98.53% (DiaretDB1), 99.18% (DrimDB) Sensitivity 100% (DiaretDB0), 99.2% (DiaretDB1), 100% (DrimDB) Specificity 98.4% (DiaretDB0), 97.97% (DiaretDB1), 98.44% (DrimDB)	Adem et al. [41]
	Colour	Kaggle (88,702), DiaretDB1 (89), and E-ophtha (107,799)	CNN	Referable/non-referable DR	AUC 0.954 (Kaggle), 0.949 (E-ophtha)	Quellec et al. [43]
	Colour	Kaggle (35,000)	CNN-ResNet34	Referable/non-referable DR	Sensitivity 85% Specificity 86%	Esfahani et al. [44]
	Colour	Messidor-2 (1748), Kaggle (88,702), and DR2 (520)	CNN	Referable/non-referable DR	Accuracy 98.2% (Messidor-2), 98% (DR2)	Pires et al. [45]
	Colour	Own dataset (30,244)	CNN (Inception V3, Inception-Resnet-V2, and Resnet152)	Referable/non-referable DR	AUC 0.946 Accuracy 88.21% Sensitivity 85.57% Specificity 90.85%	Jiang et al. [46]
	Colour	Own dataset (60,000) and STARE (131)	CNN (WP-CNN, ResNet, SeNet, and DenseNet)	Referable/non-referable DR	AUC 0.9823 (Own dataset), 0.951 (STARE) Accuracy 94.23% (Own dataset), 90.84% (STARE) Sensitivity 90.94% (Own dataset) Specificity 90.85% (Own dataset)	Liu YP et al. [47]
	Colour	DIARETDB1 (89), DIARETDB0 (130), Kaggle (15,919), Messidor (1200), Messidor-2 (874), IDRiD (103), and DDR (4105)	CNN (VGG16, custom CNN)	Referable/non-referable DR	AUC 0.786 (Kaggle, Messidor), 0.764 (Messidor-2), 0.912 (IDRiD, DDR) Accuracy 82.1% (Kaggle, Messidor), 91.1% (Messidor-2), 94% (IDRiD, DDR)	Zago et al. [48]
	Colour	Messidor-2 (1748)	CNN	Different clinical stages of DR	AUC 0.98 Sensitivity 96.8% Specificity 87%	Abramoff et al. [49]
	Colour	Kaggle (80,000)	CNN	Different clinical stages of DR	Accuracy 75% Sensitivity 30% Specificity 95%	Pratt et al. [50]
	Colour	Kaggle (2000)	DNN, CNN (VGGNET architecture), BNN	Different clinical stages of DR	Accuracy: BNN = 42%, DNN = 86.3%, CNN = 78.3%	Dutta S et al. [51]
	Colour	Kaggle (166)	CNN (InceptionNet V3, AlexNet, and VGG16)	Different clinical stages of DR	Accuracy: AlexNet = 37.43%, VGG16 = 50.03%, InceptionNet V3 = 63.23%	Wang X et al. [52]
	Colour	Kaggle (35,126)	CNN (AlexNet, VggNet, GoogleNet, and ResNet)	Different clinical stages of DR	AUC 0.9786 (VggNet) Accuracy 95.68% (VggNet) Sensitivity 90.78% (VggNet) Specificity 97.43% (VggNet)	Wan S et al. [53]
	Colour	MESSIDOR (1200)	CNN (AlexNet, VggNet16, custom CNN)	Different clinical stages of DR	Accuracy 98.15% Sensitivity 98.94% Specificity 97.87%	Mobben-ur-Rehman et al. [54]
	Colour	Own dataset (13,767)	CNN (ResNet50, InceptionV3, InceptionResNetV2, Xception, and DenseNets)	Different clinical stages of DR	96.5%, 98.1%, 98.9%	Zhang et al. [55]
	Colour	Kaggle (22,700) and IDRiD (516)	CNN (AlexNet)	Different clinical stages of DR	Accuracy 90.07%	Harangi et al. [56]
	Colour	DDR (13,673)	CNN (GoogLeNet, ResNet-18, DenseNet-121, VGG-16, and SE-BN-Inception)	Different clinical stages of DR	Accuracy 82.84%	Li T et al. [57]
	Colour	Messidor (1190)	CNN (modified Alexnet)	Different clinical stages of DR	Accuracy 96.35% Sensitivity 92.35% Specificity 97.45%	Shanthi T et al. [58]
	Colour	Own dataset (9194) and Messidor (1200)	CNN	Different clinical stages of DR	Accuracy 92.95% (own dataset) Sensitivity 99.39% (own dataset), 99.93% (Messidor) Specificity 92.59% (own dataset), 96.2% (Messidor)	Wang J et al. [59]
	Colour	Messidor (1200) and IDRiD (516)	CNN (ResNet50)	Different clinical stages of DR	AUC 0.963 (Messidor) Accuracy 92.6% (Messidor), 65.1% (IDRiD) Sensitivity 92% (Messidor)	Li X et al. [60]
AMD	Colour	407 eyes with nonadvanced AMD	DL	Distinguishes between low and high-risk AMD by quantifying drusen location, area, and size	For drusen area: ICC > 0.85; for diameter: ICC = 0.69; for AMD risk assessment: ROC = 0.948 and 0.954	Van Grinsven et al. [61]
	Colour, OCT, and IR	278 eyes with/without reticular pseudodrusen (RPD)	DL	Automatic quantification of RPD	ROC = 0.94 and 0.958; κ agreement = 0.911; ICC = 0.704	Van Grinsven et al. [62]
	Colour	2951 subjects from AREDS (834 progressors)	DL	Association between genetic variants and transition to advanced AMD	AUC: 5 years = 0.885; 10 years = 0.915	Seddon et al. [63]
	Colour and OCT	280 eyes from 140 participants	DL	Prediction of progression to late AMD	AUC = 0.85	Wu et al. [64]
	Colour and microperimetry	280 eyes from 140 participants	DL	Predictive value of pointwise sensitivity and low luminance deficits for AMD progression	AUC = 0.8	Wu et al. [65]
	Colour	> 4600 participants from AREDS	DL	Predict progression to advanced dry or wet AMD	Accuracy = 0.86 (1 year) and 0.86 (2 years); specificity = 0.85 (1 year) and 0.84 (2 years); sensitivity = 0.91 (1 year) and 0.92 (2 years)	Bhuiyan et al. [66]
	Colour	1351 subjects from AREDS (> 31,000 images)	DL	Predict progression to advanced dry or wet AMD	AUC = 0.85	Yan et al. [67]
	Colour	67,401 colour fundus images from 4613 study participants	DL	Estimate 5-year risk of progression to wet AMD and geographic atrophy based on 9-step AREDS severity scale	Weighted κ scores = 0.77 for the 4-step and 0.74 for the 9-step AMD severity scales	Burlina et al. [68]
	Colour	4507 AREDS participants and 2169 BMES participants	DL	Validation of a risk scoring system for prediction of progression	Sensitivity = 0.87; specificity = 0.73	Chiu et al. [69]
	OCT	2795 patients	DL	Prediction to nAMD within a 6-month window	AUC = 0.74 (conversion scan ground truth) and 0.886 (1st injection ground truth)	Yim et al. [70]
	OCT	671 AMD fellow eyes with 13,954 observations	DL	Predict progression to wet AMD	AUC = 0.96 ± 0.02 (3 months); 0.97 ± 0.02 (21 months)	Banerjee et al. [71]
	OCT	686 fellow eyes with non-neovascular AMD at baseline	DL	Predict conversion from non-neovascular to neovascular AMD	Drusen are within 3 mm of fovea (HR = 1.45); mean drusen reflectivity (HR = 3.97)	Hallak et al. [72]
	OCT	2146 OCT scans of 330 AMD eyes (244 patients)	DL	Predict neovascular AMD progression within 5 years	AUC = 0.74 (5 years), 0.92 (11 months), 0.86 (16 months), 0.7 (18 months), and 0.79 (48 months)	de Sisternes et al. [73]
	OCT	71 eyes of patients with early AMD and contralateral neovascular AMD (9088 OCT B-scans)	CNN	Prediction of conversion from early/intermediate to advanced neovascular AMD	AUC = 0.87 (VGG16) and 0.91 (AMDnet)	Russakoff et al. [74]
	OCT	495 eyes	DL	Predictive model to assess risk of conversion to advanced AMD	AUC = 0.68 for CNV and 0.8 for geographic atrophy	Schmidt-Erfurth et al. [75]
	OCT	2712 OCT B-scans	DL	Segmentation of features associated with AMD	Dice = 0.63 ± 0.15; ICC = 0.66 ± 0.22	Liefers et al. [76]
	OCT	930 OCT B-scans from 93 eyes of patients with neovascular AMD	CNN	Segmentation of features associated with neovascular AMD	Dice = 0.78 (IRF), 0.82 (SRF), 0.75 (SHRM), and 0.8 (PED); ICCs = 0.98 (IRF), 0.98 (SRF), 0.97 (SHRM), and 0.98 (PED)	Lee et al. [77]
RP	Colour	1128 RP and 517 healthy	CNN	Diagnose RP	AUROC 96.74%	Chen et al. [78]
	Colour	99 RP and 21 healthy	FCN	Diagnose RP	Accuracy 99.52%	Arsalan et al. [79]
RP, best disease (BD), and Stargardt	FAF	73 healthy, 125 Stargardt, 160 RP, 125 BD	CNN	Classify images into each group	Accuracy 0.95	Miere et al. [80]
Stargardt	OCT	102 healthy (33 participants) and 647 Stargardt (60 patients)	CNN	Differentiate between Stargardt and healthy	Accuracy 99.6%	Shah et al. [81]
BVMD and AVMD	FAF and OCT	118 BVMD eyes and 96 AVMD eyes	CNN	Differentiate between BVMD and AVMD	AUROC 0.880	Crincoli et al. [23]
Stargardt and *PRPH2*-related pattern dystrophy	FAF	304 Stargardt (40 patients) and 66 *PRPH2* (9 patients)	CNN	Differentiate between Stargardt and *PRPH2*-related pattern dystrophy	AUROC 0.890	Miere et al. [82]
*ABCA4*-, *RP1L1*-, and *EYS*-related retinopathy	OCT	58 IRD and 17 healthy	DL	Predict causative gene	*ABCA4* 100% accuracy; *RP1L1* 66.7 to 87.5%; *EYS* 82.4 to 100%; healthy 73.7 to 100%	Fujinami-Yokokawa et al. [83]
Stargardt disease	FAF	47 images (24 patients)	CNN	Segment flecks	Dice score: 0.54 ± 0.14 for diffuse speckled patterns; 0.71 ± 0.08 for discrete flecks	Charng et al. [84]
Stargardt disease and AMD	FAF	320 healthy 320 AMD and 100 Stargardt	CNN & FCN	Detect and segment atrophy	Atrophy screening: AMD 0.98 accuracy; Stargardt 0.95 Segmentation: AMD overlapping ratio of 0.89 ± 0.06; Stargardt: 0.78 ± 0.17	Wang et al. [85]
Stargardt and pattern dystrophy	FAF	110 AMD, 204 Stargardt, and pattern dystrophy	CNN	Differentiate between AMD and IRD-associated macular atrophy	AUROC 0.981	Miere et al. [86]
Stargardt disease	OCT	87 scan sets (22 patients)	FCN	Detect outer and inner limits of the retina	Mean difference: 2.10 µm and 0.059 mm^3^ in central macular thickness and volume between model and annotators	Kugelman et al. [87]
	AOSLO	142 controls and 148 Stargardt	FCN	Identify cone photoreceptors	Dice score: 0.9431 ± 0.0482	Davidson et al. [88]
RP and CHM	OCT	300 B-scans with RP and 300 with CHM	FCN	EZ segmentation	Similarity of 0.894 ± 0.102 automatic vs manual grading for RP; 0.912 ± 0.055 for CHM	Camino et al. [89]
CHM	OCT	16 eyes CHM and 5 healthy	Nonneural (RF)	EZ segmentation	0.876 ± 0.066 Jaccard similarity index	Wang et al. [90]
*USH2A*-related RP	OCT	126 volume scans (126 patients)	CNN	EZ segmentation	Dice score 0.79 ± 0.27	Loo et al. [91]
	OCT	86 volume scans (86 patients)	CNN	EZ segmentation	Dice score 0.867 ± 0.105	Wang et al. [92]
RP	OCT and IR	2918 (314 patients)	CNN and FCN	Predict VA below or above 20/40	AUROC 0.85	Liu et al. [93]
Blue cone monochromacy (BCM)	OCT	26 IRD, 16 BCM, 3 normal (patients)	Nonneural	Predict foveal sensitivity and VA	0.174 RMSE for VA and 2.91 for sensitivity	Sumaroka et al. [94]

*RP*, retinitis pigmentosa; *CNN*, convolutional neural network; *AUROC*, area under the receiver operating characteristic; *FROC*, free-response receiver operating characteristics; *ICC*, intraclass correlation coefficients; *FCN*, fully convolutional network; *FAF*, fundus autofluorescence; *OCT*, optical coherence tomography; *BVMD*, best vitelliform macular dystrophy; *HR*, hazard ratio; *AVMD*, adult-onset vitelliform macular dystrophy; *DL*, deep learning; *AMD*, age-related macular degeneration; *IRD*, inherited retinal dystrophy; *AREDS*, age-related eye disease study; *BMES*, Blue Mountains Eye Study; *AOSLO*, adaptive optics scanning laser ophthalmoscopy; *CHM*, choroideremia; *EZ*, ellipsoid zone; *VA*, visual acuity; *RMSE*, root-mean-square error; *IRF*, intraretinal fluid; *SHRM*, subretinal hyperreflective material; *PED*, pigment epithelial detachment; *SRF*, subretinal fluid

Wong et al. [96] developed a model to classify DR stages based on microaneurysms and haemorrhages, while others used exudates, blood vessel mapping, and the optic disc. [97, 98] The sensitivity of automatic DR screening has been reported as ranging from 75 to 94.7%, with comparable specificity and accuracy [99]. Several publicly available retinal datasets have been used to train, validate, and test these AI systems, and also to compare performance against other systems; namely, DIARETDB1, Kaggle, E-ophtha, DDR, DRIVE, HRF, Messidor, Messidor-2, STARE, CHASE DB1, Indian Diabetic Retinopathy Image Dataset (IDRiD), ROC, and DR2 [57, 100–108]. Several studies have used these datasets to detect red lesions, microaneurysms, DR lesions, exudates, individual DR stages, and blood vessel segmentation [38, 40, 41, 43, 52, 109, 110].

Another area of focus is the detection of DMO, currently assessed by OCT as the gold standard. AI-based groups have tried detecting DMO from colour fundus photography based on exudates and accurate identification of the macula. Automated detection via OCT imaging is ongoing, focusing on retinal layer segmentation [111, 112] and specific lesion (e.g. cysts) identification [113–118]. Recently, DL has also been used to detect macular thickening based on colour photographs, and it has been found to be comparable to OCT-measured thickness [119].

Multiple programs have tried to use AI-based methods in population-based screening for DR. The United States Food and Drug Administration (US FDA) has recently approved IDx-DR, a CNN for screening DR stages in adults aged 22 years or older [49, 120]. Initial versions of IDx-DR have been evaluated as part of the Iowa Detection Programme and have shown good results in White, North African, and Sub-Saharan populations [25]. Similar software, like the RetmarkerDR in Portugal and EyeArt in Canada, have been tested in local screening programs [121, 122]. Multiple South-Asian eye institutes are also involved in development and validation of AI-based algorithms in DR [95, 123, 124]. Recently, a Singapore-based DL tool has shown comparable diagnostic accuracy to manual grading, and a semi-automated DL model involving a secondary human assessment may prove to be the most cost-effective model [125, 126]. Their real-world performance remains to be tested [127].

### Age-related macular degeneration (AMD)

The use of AI with DL tools has great potential in AMD, both for diagnostic purposes—while allowing for a more efficient and accurate approach—to prognostication of affected individuals and perhaps to directly determine (predict) efficacy of treatments. The most common imaging modalities being explored in the field of AI for AMD are OCT, colour fundus image, and fundus autofluorescence (FAF). OCT-angiography (OCTA) has also been used in DL approaches to diagnose and classify AMD, achieving high accuracy and sensitivity [128, 129]. Due to the huge number of studies, selected key ones will be discussed, with a summary of a broad range of studies in Table 1.

One of the first attempts to evaluate ML algorithms in risk assessment of AMD was a European study by van Grinsven et al. that aimed to detect and quantify drusen on colour fundus photographs in eyes without and with early to moderate AMD [61]. This study demonstrated that the proposed system was in keeping with experienced human observers in detecting the presence of drusen as well as estimating the area, with an ICC greater than 0.85. For AMD risk assessment, it achieved a ROC of 0.948 and 0.954—similar performance to human graders. Subsequently, the same group explored another algorithm for automatic detection of reticular pseudodrusen (RPD) [62]. This followed a multimodal imaging approach using colour fundus, FAF, and near-infrared images, with automated quantification having similar performance to the observers.

In 2018, Schmidt-Erfuth et al. evaluated the predictive potential of ML in terms of best-corrected visual acuity (BCVA) by analysing OCT volume scan features—intraretinal fluid (IRF), subretinal fluid (SRF), and pigment epithelial detachment (PED) [130]. A modest correlation was found between BCVA and OCT at baseline (*R*^2^ = 0.21), while functional outcome prediction accuracy increased in linear fashion. The same group then explored automated quantification of fluid volumes using a DL method and a CNN, using OCT data from the HARBOR study (NCT00891735) for neovascular AMD (nAMD) [131]. Retinal fluid volumes (IRF, SRF, and PED) were then validated by the authors as important biomarkers in nAMD [132].

A more recent study attempted to introduce an AI system that combines 3D OCT images and automatic tissue maps in individuals with unilateral nAMD to predict progression in the contralateral eye [70]. It achieved a sensitivity of 80% at 55% specificity and 34% specificity at 90% sensitivity while being able to identify high-risk groups and changes in anatomy before conversion to nAMD, outperforming 5 out of 6 experts. Also, the age-related eye disease studies (AREDS and AREDS2) used DL algorithms and survival analysis to predict risk of late AMD, which achieved high prognostic accuracy [133].

Several segmentation models have been described in AMD. In 2018, De Fauw et al. created a landmark OCT image segmentation model that utilised a DL framework to perform segmentation and automated diagnosis of retinal diseases [134]. Subsequently, Liefers et al. validated a DL model for segmentation of retinal features specifically in individuals with atrophic AMD and nAMD, with results comparable to independent observers [135]. A further automated segmentation algorithm with a CNN has been explored to quantify IRF, SRF, PED, and subretinal hyperreflective material (SHRM) in nAMD [136]. There was good agreement for both the segmentation and detection of lesions between clinicians and the network (dice scores ≥ 0.75 for all features). Two applications with validated automated DL segmentation algorithms are currently commercially available: RetinAI (Medical AG, Switzerland) and RetInSight (Vienna, Austria) [137].

Dry AMD with geographic atrophy (GA) has also been actively investigated. Zhang et al. developed a DL model that segments and classifies GA on OCT images, achieving similar performance to manual specialist assessment [138]. Another group segmented GA in both OCT and FAF images and had reasonable agreement, with better performance (highest dice) in FAF [139]. GA algorithms have also been used to predict VA, with certain features such as photoreceptor degeneration having high predictive significance [140].

### Inherited retinal disorders (IRD)

AI algorithms using multimodal imaging techniques have been developed to facilitate the diagnosis [78], classification [80], decipher the genetic aetiology [83], and measure the progression rate of IRD [89, 84].

Chen et al. have developed a CNN that detects if a patient has retinitis pigmentosa (RP) by analysing colour fundus images, with an overall accuracy of 96% (versus 81.5% from four ophthalmology experts) [78]. Another group proposed an FCN that detects pigment in colour images and diagnoses RP with an accuracy of 99.5% [79].

To predict aetiologies, Miere et al. have created a CNN model that can distinguish between FAF images from patients with Stargardt disease (STGD), RP, and best disease (BD), with an overall accuracy of 0.95 [80]. Furthermore, Fujinami-Yokokawa et al. used OCT images to predict causative genes (*ABCA4*, *RP1L1*, and *EYS*) through a DL platform [83]. They achieved an accuracy of 100% for *ABCA4*, 66.7 to 87.5% for *RP1L1*, 82.4 to 100% for *EYS*, and 73.7 to 100% for healthy control images. Miere et al. also created a CNN that is able to outperform specialists in distinguishing between FAF images of STGD and *PRPH2*-related macular dystrophy (AUROC 0.890 versus experts 0.816) [82]. Shah et al. also achieved an accuracy of 99.6% with a model distinguishing between OCT images from patients with STGD and controls [81]. Crincoli et al. combined image processing with a CNN to differentiate between BD and adult-onset vitelliform macular dystrophy using FAF and OCT images, with an AUROC of 0.880 [23]. Moreover, this endeavour has been recently markedly upscaled by Pontikos et al. to differentiate between 36 gene classes by exploiting multimodal imaging [141]. However, further development is needed, given more than 300 genes are known to cause IRD to date.

STGD is the most prevalent inherited macular dystrophy, and it can affect both children and adults, with multiple ongoing clinical trials [142]. Charng et al. developed a CNN algorithm that segments flecks and is able to monitor their progression over time [84]. They obtained an overall agreement between manual and automatic segmentation of 0.54 ± 0.14 dice score for diffuse speckled patterns and 0.71 ± 0.08 for discrete flecks. Wang et al. also used FAF images, detecting and quantifying areas of atrophy in STGD and AMD [85]. They obtained an accuracy of 0.98 for differentiating normal eyes from those with AMD-related atrophy and 0.95 for eyes with STGD. Atrophic areas were also segmented manually and automatically, with an overlap ratio of 0.89 ± 0.06 in AMD and 0.78 ± 0.17 in STGD [85]. Miere et al. also assessed atrophy and developed a CNN that differentiates between FAF images with GA secondary to AMD and IRD-associated, with an AUROC of 0.981 [86].

Automatic macular OCT segmentation by the device manufacturers is often inaccurate in IRD, requiring manual correction in over one-third of scans [143]. OCT images of STGD were used to create an improved DL-based algorithm that is able to segment the inner and outer retinal limits, providing faster and better macular thickness and volume quantification [87]. Lastly, adaptive optics scanning light ophthalmoscopy images of STGD have also been used to develop an FCN that is able to accurately count macular cones (dice score: 0.9431 ± 0.0482) [88].

Other tools are being designed to assess disease severity and potentially have applications in determining eligibility for interventional trials. A CNN has been developed by Camino et al. that segments preserved EZ area on OCT images from patients with RP and choroideremia (CHM) [89]. This tool reached 0.894 ± 0.102 similarity between automatic and manual grading for RP and 0.912 ± 0.055 for CHM. Loo et al. also targeted EZ segmentation and validated their algorithm for macular telangiectasia in patients with *USH2A*-related RP, with excellent applicability (dice score 0.79 ± 0.27) [91]. Similarly, Wang et al. also tested an EZ segmentation CNN in *USH2A*-RP and obtained a Dice score of 0.867 ± 0.105 [92]. CHM EZ segmentation was then attempted by Wang et al. through a nonneural random forest approach and reached a Jaccard similarity index between manual and automated segmentation of 0.876 ± 0.066 [90].

Predicting VA based on OCT and infrared images in RP has been assessed by Liu et al*.* They were able to determine if a patient with RP had VA below or above 20/40, with an AUC of 0.85 [93]. Sumaroka et al. also developed a nonneural network to predict foveal sensitivity (Humphrey visual field testing), VA, and possible outcome of therapy in patients with blue cone monochromacy based on OCT scans, with good results [94].

### Retinopathy of prematurity (ROP)

ROP is an important cause of preventable childhood blindness worldwide [144]. ROP causes abnormal blood vessel growth and can be detected by trained ophthalmologists using indirect ophthalmoscopy, with access to adequate, timely screening potentially limited due to the requirement of highly trained personnel and equipment. DL-based detection and staging of ROP[145] by evaluation of posterior pole fundus images has been attempted with high sensitivity and specificity [146]. Authors have developed a ROP vascular severity score with good correlation with the labels set by the International Classification of Retinopathy of Prematurity committee [147]. The DeepROP score [148] and i-ROP DL system are DL algorithms developed to evaluate clinically significant severe ROP at the posterior pole [149]. ROP plus disease, a more aggressive form of ROP, is often difficult to diagnose given the lack of consensus among ophthalmologists; several authors have evaluated automated algorithms that may be able to objectively diagnose plus disease [150–153].

These study limitations are the review of the literature in a non-systematic approach, possibly leading to some papers being omitted or not adequately prioritised; and editorial restrictions, which prevented us from doing a comprehensive review of AI applications in all retinal disorders. Substantial research has been undertaken in other fields of medical retina (e.g., uveitis and oncology), which will be reviewed in a subsequent project. [154, 155]

## Concluding remarks and future directions

Retinal disease has been at the forefront of AI in ophthalmology, with the first AI-related publication being on DR. Since then, research groups focusing their efforts on AI have multiplied around the world, targeting all aspects of the patient journey, including diagnosis, triage, and prognostication, by leveraging multiple imaging (and functional) modalities, as well as a range of AI tools. A shortage of medical professionals is anticipated in the short term, likely further increasing healthcare inequalities and challenging our ability to improve care for preventable diseases [156]. AI represents one important approach to help meet these challenges and moreover facilitate improvements in patient care—both at the individual level with more timely, accurate, and bespoke management, as well as population-level, large-scale healthcare. Ever-improving DNN and CNN algorithms can become a helping hand for healthcare to lean on towards meeting current capability endpoints.

Despite the huge promise, many challenges remain for AI in ophthalmology, including, (i) the need for larger, more diverse, and representative datasets that fully represent real life, (ii) the closer collaboration by experts (both national and international) to develop disease-specific consensus and subsequently provide a comprehensive large volume of image grading, and (iii) greater synergy between healthcare professionals, patients, and data scientists, communicating and improving the software interface as it is being iteratively created, and ensuring it complements the human interaction that underpins the practice of medicine, rather than seeking to replace it [157]. Further uses of AI are yet to be explored, such as multimodal inputs to determine the best candidates for interventional clinical trials, the selection of the ideal anti-VEGF and therapeutic scheme in nAMD, and the estimation of functional impairment based on structural parameters for IRD, among others.

The future of healthcare will increasingly incorporate the advantages that AI can provide to improve the lives of our patients and no doubt perform assessments quicker and more accurately than retina specialists can currently sustainably provide, allowing us to spend more time being better clinicians and scientists. Nevertheless, as always with new technology, there will be new learnings and surprises along the way.
